# What is a High-Quality Moral Case Deliberation?-Facilitators’ Perspectives in the Euro-MCD Project

**DOI:** 10.1007/s10730-023-09519-w

**Published:** 2024-02-05

**Authors:** Lena M. Jakobsen, Bert Molewijk, Janine de Snoo-Trimp, Mia Svantesson, Gøril Ursin

**Affiliations:** 1 Department of Health and Care Sciences, Faculty of Health Sciences, UiT The Arctic University of Norway, Harstad, Norway; 2 Department of Ethics, Law and Humanities, Amsterdam UMC, VU University, Amsterdam, The Netherlands; 3 Center of Medical Ethics, Institute of Health and Society, University of Oslo, Oslo, Norway; 4 Department of Ethics, Law and Humanities, Amsterdam UMC, VU University, Amsterdam, The Netherlands; 5 Faculty of Medicine and Health, University Health Care Research Centre, Örebro University, Örebro, Sweden; 6 Faculty of Nursing and Health Science, Nord University, Bodø, Norway

**Keywords:** Euro**-**MCD, Facilitator, Clinical ethics, Reflection groups, Ethics training, Ethics support

## Abstract

The evaluation of the European Moral Case Deliberation Outcomes project (Euro-MCD) has resulted in a revised evaluation instrument, knowledge about the content of MCD (moral case deliberation), and the perspectives of those involved. In this paper, we report on a perspective that has been overlooked, the facilitators’. We aim to describe facilitators’ perceptions of high-quality moral case deliberation and their Euro-MCD sessions. The research took place in Norway, Sweden, and the Netherlands using a survey combined with interviews with 41 facilitators. Facilitators’ perceived that attaining a high-quality MCD implies fostering a safe and respectful atmosphere, creating a wondering mode, being an attentive authority, developing moral reflective skills, reaching a common understanding, and ensuring organisational prerequisites for the MCD sessions. Our central conclusion is that efforts at three levels are required to attain a high-quality MCD: trained and virtuous facilitator; committed, respectful participants; and organizational space. Furthermore, managers have a responsibility to prepare MCD participants for what it means to take part in MCD.

## Introduction

Healthcare professionals frequently encounter ethical challenges in the course of their work, which necessitates the exploration of different approaches to support ethical decision-making. Clinical ethics consultant services (CESS), clinical ethics committees, and moral case deliberation (MCD) are among the methods employed to assist healthcare professionals in addressing these challenges (Rasoal et al., 2017a, 2017b). MCD, a specific type of clinical ethics support, involves healthcare professionals coming together in a group setting facilitated by an expert to collectively examine ethically complex patient cases using a structured conversation (Metselaar et al., 2015; Molewijk et al., 2008; Stolper et al., 2016). MCD is recognised as a bottom-up approach as its focus on concrete deliberation helps professionals contextualise the ethical aspects of the case and allocate moral responsibility (Rasoal et al., 2017a, 2017b).

The primary objective of MCD is to improve the quality of care provided (Abma et al., 2009; Silén & Svantesson, 2019). To achieve this goal, it is crucial to understand the processes involved in MCD to effectively support professionals in dealing with ethically challenging situations (Heidenreich et al., 2017; Inguaggiato et al., 2019; Rasoal et al., 2016). Furthermore, evaluation research is essential for the professionalisation and improvement of MCD’s quality while enhancing its practical utility (Schildmann et al., 2013). Although evaluation research has demonstrated the impact of MCD on practice (Hem et al., 2018; Janssens et al., 2015), the complex psychosocial nature of MCD makes it difficult to establish objective evidence and empirical endpoints (Haltaufderheide et al., 2022; Haan et al., 2018).

To measure the perceived importance and experienced outcomes of MCD, the European Moral Case Deliberation Outcomes Instrument (Euro-MCD) was developed (Svantesson et al., 2014). This instrument has since undergone revision and evolved into Euro-MCD 2.0, featuring fewer and more abstract domains, namely moral competence, moral teamwork, and moral action (de Snoo-Trimp, de Vet, et al., 2020). A field study revealed variations in moral reflexivity, moral attitude, and emotional support among different countries (Svantesson et al., 2019). Moreover, an analysis of Swedish audio recordings of MCD sessions revealed variations in the extent of moral reflection across different workplaces and facilitators, with some workplaces predominantly focusing on the psychosocial work environment (Svantesson et al., 2018). Consequently, we have turned our attention to examining the influence of facilitators on MCD to gain a better understanding of the findings from the Euro-MCD project as well as to explore facilitators’ perceptions of high-quality MCD. Given the pivotal role of facilitators in fostering quality dialogue, our aim is to elucidate facilitators’ descriptions of high-quality MCD and their Euro-MCD sessions.

### Study Background

In the Netherlands, the setting for the moral case deliberations was psychiatric facilities, facilities for people with mental disabilities, and hospitals. In Norway, the setting was municipal care, and in Sweden, it was municipal and hospital care. Each country had different ways of organising MCD within the Euro-MCD project. In the Netherlands, the MCDs were planned according to a structured organisation of an MCD (Dauwerse et al., 2014) with certified facilitators using a reflection model (Stolper et al., 2016). The most common conversation methods were the dilemma method, the Socratic dialogue, and the “Utrechtse” or “Nijmeegse” method (Molewijk, 2014; Stolper et al., 2015). In Norway, the MCDs were organised within a national project in municipal care and named “ethics reflection groups” (2007–2015; Magelssen et al., 2016). The facilitators were trained in the CME (Centre for Medical Ethics) method (Magelssen et al., 2016), a six-step guide for structuring ethical reflection: (1) defining the ethical problem; (2) describing the facts of the case; (3) stating the views of the involved parties; (4) discussing the values, laws, and guidelines relevant to the problem; (5) considering the different courses of action; and (6) performing an overall assessment (Lillemoen & Pedersen, 2015).

In Sweden, there was no consensus on models, although the dilemma method had been implemented in a few hospitals (Silén & Svantesson Sandberg, 2022); a Swedish research team is now working towards a national consensus on models. The MCDs departed from local or individual facilitator style, but, nonetheless, a process was detected of promoting security and well-being, helping to navigate moral reflections, challenging homogeneity, accommodating to needs, and steering authority and expertise (Rasoal, Kihlgren 2016).

## Methods

This study is part of the broader Euro-MCD Project (de Snoo-Trimp, de Vet, et al., 2020; de Snoo-Trimp, Molewijk, et al., 2022; de Snoo-Trimp et al., 2019; de Snoo‐Trimp et al., 2017; Heidenreich et al., 2017; Rasoal et al., 2017a, 2017b; Silén & Svantesson, 2019; Snoo‐Trimp et al., 2017; Svantesson et al., 2019; Svantesson et al., 2018).

### Design

This study applies a qualitative design employing structured interviews. Qualitative research approaches a scenario by studying things in their natural setting by interpreting or making sense of people’s perceptions of a phenomenon’s meaning to gain a broader understanding of the world (Denzin & Lincoln, 2011, p.3).

### Questionnaire

We constructed a study-specific questionnaire comprising closed- and open-ended questions. Nine closed-ended questions were used to gather demographic data and descriptions of the project’s MCD sessions. An opportunity for comments was given as an attachment to all closed-ended questions. Three open-ended questions concerned high-quality MCD, including essentials, difficulties in facilitating, and the very aim of MCD. The questionnaire was pilot-tested on three facilitators.

### Data Collection

The data collection process varied across the three countries. In the Netherlands, the questionnaire was distributed online through Survalyzer, while in Sweden, it was distributed via email. However, in Norway, the initial data collection using the questionnaire faced challenges as some survey questions did not yield sufficient depth in the responses. Consequently, the data collection approach in Norway was adjusted, and structured telephone interviews were conducted instead to elicit more comprehensive insight. Additionally, to augment the Swedish data, secondary data from a previous study involving the same Swedish facilitators was incorporated. The use of this secondary data was approved by Rasoal (Rasoal et al., 2017a, 2017b).

### Data Analysis

We analysed the closed-ended responses via descriptive statistics using the Statistical Package for the Social Sciences (SPSS), version 22. For the analysis of the open-ended written responses, comments, and interview transcripts, we used content analysis (Graneheim and Lundman, 2004), focusing on the perspective of high-quality MCD.

Initially, each author independently conducted iterative readings of the text in their respective languages to gain a comprehensive understanding. After the initial reading, all text was translated into English (a second language for the authors) and cross-checked for linguistic accuracy by the respective authors. LJ then identified meaningful units and condensed them into codes at the descriptive level. To ensure accuracy, the authors collectively reviewed the translations from meaning units to codes and made necessary adjustments. The next step involved categorising all codes in a joint table based on their shared content. These categories were then abstracted to derive preliminary and potential themes. At this stage, the focus was on categorising the underlying meanings from the data together with preliminary descriptive themes (Bengtsson, 2016). Given the nature of the data, the level of abstraction was low, and the interpretation degree was closer to a manifest level than a latent one (Graneheim et al., 2017).

Throughout the process, frequent meetings were held in which codes, categories, and themes were collectively discussed among all co-authors. These discussions led to the renaming and repositioning of codes and categories as well as adjustments to the naming of themes. Recategorisations and reformulations continued until the final report was authored.

### Ethical Considerations

The Institutional Review Board of each country was contacted for this Euro-MCD project. In Sweden, the Swedish Regional Ethical Review Board provided an advisory statement that they had “no objection to this study” (DNR, 2012, p. 34). The Norwegian Centre for Research Data (NSD) required no formal IRB approval. In the Netherlands, the Ethical Review Board of the Free University of Amsterdam stated that further ethical approval was unnecessary (2017.612) according to the Dutch Medical Research Involving Human Subjects Act (WMO). All MCD facilitators received an informational letter regarding voluntary participation, pseudo-anonymisation of data, and their right to withdraw themselves and the data collected about them without providing a reason. Informed consent was obtained either orally or through e-mail.

## Findings

### Participants

All 66 facilitators who initially participated in the Euro-MCD project were invited (33 from the Netherlands, 22 from Norway, and 11 from Sweden), of whom 41 participated in the study (Table 1) for an overall response rate of 62% (the Netherlands 66%; Norway 36%; Sweden 100%). Changes in the workplace or no response to the invitation mail were cited as reasons for non-response. The Euro-MCD project facilitator group consisted of a wide range of professionals with experience in facilitating and training (Table 1).

**Table 1:** Characteristics of facilitators in the Euro-MCD project

	Total	Netherlands	Norway	Sweden
Total	41	22	8	11
Response rate: (/total)	62% (/66)	66% (/33)	36% (/22)	100% (/11)
Female (%)	23 (56)	12 (55)	7 (88)	4 (36)
Age: mean (range)	50 (24–73)	46 (24–61)	47 (37–55)	61 (36–73)
**Main professional role**				
Chaplain/deacon	11	5	0	6
Philosopher	7	4	1	2
Nurse	9	3	4	2
Physician	2	1	0	1
Psychologist/psychotherapist	5	3	2	0
Nurse manager/policymaker	4	3	1	0
Teacher	3	3	0	0
**Experience as facilitator**				
Less than 3 years	22	17	8	4
More than 3 years	10	5	0	7
Trained as facilitator (%)	33 (87)	22 (100)	7 (88)	0
Certificate yes/total	27/33	21/22	5/7	0
**Total number of facilitated MCD**				
Fewer than 5 sessions	7	5	1	1
5–10 sessions	13	7	1	5
More than 11 sessions	20	9	6	5

### Descriptions of the Euro-MCD Sessions

The sessions (Table 2) were described as a dialogue on real patient/family situations for a minimum of one hour to determine the correct action to take. On average, the sessions lasted 80 minutes (range 45–120 minutes). Most sessions included five to ten participants. Though real patient and family situations were typically used, hypothetical cases or general themes/topics and collaboration among healthcare professionals in the workplace were also discussed. Most facilitators reported that the session participants ultimately agreed on a solution regarding the case they were discussing. However, half of the facilitators indicated that they as facilitators were sometimes normative and provided advice regarding the best action to take.

**Table 2:** Descriptions of the Euro-MCD sessions, *n* (%)

Length of MCD session	*N (mean)*	Arriving at a conclusion	*N* (%)
45–60 min	13 (32)	What is right and what action to take	28 (83)
70–90 min	19 (46)	Insight into the complexity of the situation	1 (2)
More than 90 min	8 (20)		
Number of participants		How to manage a recurring situation	1 (2)
Fewer than 5	2 (5)		
5–10	31 (76)	What to stand for as a team	1 (2)
11–15	6 (15)	No conclusion	3 (7)
Most discussed topics^1^		Being normative as facilitator	
A real patient situation	17 (41)	Yes	2 (5)
Family in a real situation	16 (39)	Sometimes	21 (51)
Work environment	6 (15)	No	17 (41)
Team collaboration	8 (20)		
Other	2 (5)		
		^*1*^*Respondents could indicate* ≥ *1 response option*	

### Perceptions of High-Quality MCD

Facilitators perceived that for a high-quality MCD to occur, they were required to foster a safe and respectful atmosphere, create a wondering mode, be an attentive authority, develop ethical and reflective skills, reach a common understanding, and ensure organisational prerequisites.

#### Fostering a Safe and Respectful Atmosphere

Mutual respect regarding different opinions, thoughts, and equality among participants and eliminating patriarchy in clinical practices were perceived as crucial. These actions implied an open dialogue of mutual contribution, leaving behind negative thoughts and experiences, and treating each other respectfully and fairly, which can be hard:

Sometimes it is difficult to create an open and secure atmosphere in a team due to hierarchy; for instance, because nurses look up to doctors, they cannot listen well to each other, or they do not dare to speak up. (Dutch facilitator)

It all comes down to how the manager practices leadership, the persons in the group, and their relations. Sometimes it works perfectly, and sometimes it doesn’t. (Swedish facilitator)

Facilitators stated that when participants succeeded, the atmosphere became secure, and everybody felt free to speak their minds. This was viewed as the collective responsibility of both the facilitators and the participants; the participants were also expected to contribute to creating a safe and open environment. Promoting a safe atmosphere was perceived as necessary for facilitating equality and ensuring that everyone’s viewpoints and thoughts were counted and valued.

It is crucial to manage the balance of all the different views as there may be participants who very blatantly manifest their opinions and colour the meeting, shouting loudly, and somehow promoting themselves… (Norwegian facilitator)

The facilitators felt they needed to have control of the group to help the participants alternate between rational thinking and emotions in their judgements while maintaining a respectful dialogue. Fostering a “*trusting*” atmosphere was also perceived as one of the main goals of MCD. When this occurs, the trust built within the group can also expand to encompass the other staff and patient interactions.

#### Creating a Wondering Mode

Respondents emphasised that conducting a high-quality MCD served to open minds and hearts through others’ perspectives. Facilitators wanted the participants to be curious and willing to ask questions; to do so, the participants needed a positive and open-minded attitude when joining the sessions. Here, participants were required to understand the purpose of moral deliberation to create an atmosphere of curiosity. When participants understood this, facilitators felt that they could begin focusing on quality deliberations.

In the end, I think they understood that it is not about making it academically complicated; it is about something we want to participate in for the benefit of those they care for in their job, and that’s when they became motivated… (Norwegian facilitator)

Facilitators found it challenging when the participants seemed mentally absent; they were then *just a group of unmotivated and unwilling participants…*(Dutch facilitator). Consequently, they described the need for secure and relaxed participants who focused on each other and the case or issue at stake to create an atmosphere of inquisitiveness. Additionally, some facilitators perceived it as important that they were coming from outside the clinical context, making allowances to ask probing questions from an unknowing position:

I don’t come and lecture them on ethical principles and so on; I am not their teacher… but instead, I let the discussion float in the group and just try to be listening. Since I am an outsider, I use the opportunity to ask naïve questions and allow them to talk. (Swedish facilitator)

#### Being an Attentive Authority

Good organisational skills were perceived as crucial, which implies the need to maintain structure and ensure proper time management for the dialogue to progress. For some facilitators, following a clear stepwise conversation plan and dialogue rules was rendered essential.

Keeping the focus on the steps and having a good structure and clear adherence to the rules of conduct are important for having a good MCD session. (Dutch facilitator)

There were different perceptions regarding the responsibility to provide a worthwhile case or issue to review as to whether the participants should offer a relevant case or the facilitator should be prepared with one. Other facilitators stressed the importance of being secure in handling conversations and enabling the participants’ contributions:

I am very careful about maintaining the order of speech, and some people find it difficult to accept. They want to interrupt and quickly have their say; they find waiting for their turn difficult. So, there is an educational problem I experience in some groups. Some can sit quietly every time, and I will then have to bring them into the conversation. (Swedish facilitator)

Furthermore, some facilitators desired traits such as empathy, patience, engagement, and knowledge of and experience in ethics, law, the healthcare system, and ward functions. Other facilitators described their experiences of facilitating as overwhelming, and they were fearful of not having all the answers.

#### Developing Ethical and Reflective Skills

Reflective skills were perceived as necessary, and the ability to recognise moral questions or ethical issues was important to cultivate. Once identified, a solution can typically be found.

I try to get them to articulate the ethical issue more clearly. There are many issues, such as psychological, legal, and medical problems. However, this is about finding ethical components in the issues and drawing them out. (Swedish facilitator)

There are a lot of easy questions you can ask, so they become apparent in the group without you having to answer them. (Swedish facilitator)

Promoting an understanding of different perspectives on ethical issues was considered vital, enabling participants to broaden their perspectives. In this regard, the facilitators ensure a process of examining values, opinions, and existential issues with a reflective attitude toward one’s thoughts as well as an investigation by asking questions of others:

The ability to make a well-considered decision in the case of a concrete moral dilemma is based on a joint reflective learning process in which sharing perspectives and complementary expertise is central. (Dutch facilitator)

The facilitators clarified that the problem-solving process was based on participants’ reflective attitudes toward their and others’ values and thoughts. Therefore, facilitators found it essential to help the participants structure the content by basing the dialogue on examining values and opinions. Insights gained from the sessions were described as an overall aim of MCD and using reflective skills to make thoughtful decisions.

I asked the staff whether they found it useful, and they were excited. One told of when he recognised a dilemma we had discussed on the night shift, and that was just the impact we wanted … analysis of the dilemma. (Norwegian facilitator)

#### Reaching a Common Understanding

Reaching a shared understanding of the most suitable decision, if possible, was seen as vital by most of the facilitators. Particularly, the process of group dialogue was also important, including the process of understanding:

We gain a common understanding and an understanding of how the other person thinks and how we can solve ethical challenges together. Interaction between staff will become better… (Norwegian facilitator).

It is not about determining right or wrong; we can make reflections, try to use them, and make an agreement together. (Swedish facilitator).

Achieving consensus was expressed as enabling a better understanding of other people’s values and moral concerns, which may help participants interact more comfortably with patients and families. The participants can also feel secure in providing grounded choices for actions in an ethically challenging situation. Many of the facilitators stated that MCD participants should try to find a shared understanding and common ground concerning actions that are believed to be morally acceptable among all the stakeholders of the ethical issue. When discussing a real case, the group reached a conclusion after providing advice for action, realising one of the aims of a high-quality session, according to some informants. Many facilitators emphasised how the process of recognising the value of other participants’ points of view could contribute to a better work climate:

When we make a moral inquiry from cases in our daily practice, we can agree on how much room we have to manoeuvre. This makes the working day less noisy, and we learn to respect and know each other better. (Dutch facilitator)

A high-quality session would give the participants a new understanding and acceptance of their co-workers and an appreciation of the patient’s point of view.

#### Ensuring Organisational Prerequisites

Feeling enabled and supported by managers in organisations was described as crucial for facilitating high-quality MCDs. However, one facilitator felt that it was difficult to gain the support of organisation management for MCD:

The management is a large group of people. They are responsible for the departments they are running to ensure enough staff and so on, and then they need to allow the staff time to participate in something that they think leads to nothing? So, I have to constantly motivate the management at different levels and help them understand that this is important and meaningful. (Swedish facilitator)

Some facilitators felt anxious that making the MCD session feasible was too much pressure, as they were trying to facilitate the MCD session during a hectic time in their ward. In this, they experienced little support from first-line managers:

Ethical reflections can quickly become downgraded when there is poor staffing, and one feels that it comes at the expense of patients. I guess it’s the challenge we struggle with in everyday life to get it done. (Norwegian facilitator)

Facilitators perceived the physical presence of the staff directly involved in the cases as crucial to be able to prioritise the MCD. Having sufficient time for in-depth dialogue was a concern, and the lack of spaces with privacy or interruptions during MCD hampered this: ‘It is difficult when the setting is chaotic—for instance, at the acute ward where people walk in and out, and phones are continuously ringing’ (Dutch facilitator). Facilitators stated that when this happened, the quality of the MCD was impaired.

## Discussion

**Fig. 1:**
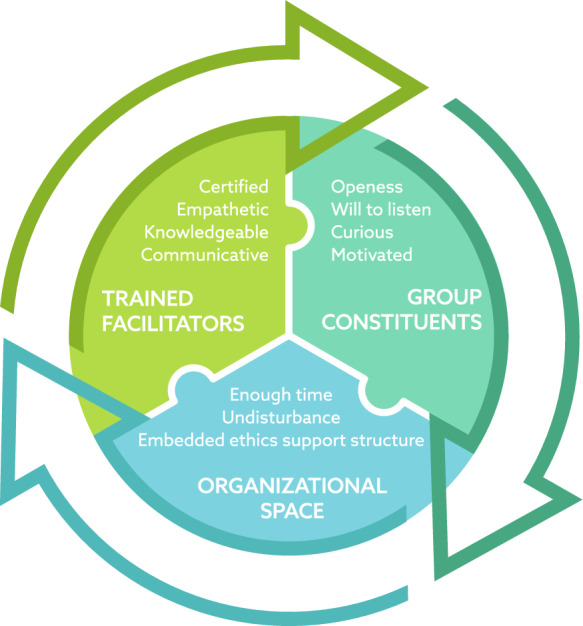
The dynamic interplay of facilitator, participants and organisation

Facilitators in our study perceived a high-quality MCD to emerge from fostering a safe and respectful atmosphere, creating a wondering mode, being an attentive authority, developing ethical and reflective skills, reaching a common understanding, and ensuring organisational prerequisites. In the following discussion, we delve into how to achieve high-quality MCD results through the intricate interaction of the following three key factors: The training and competencies of the facilitator, the commitment and respect of the MCD participants, and the responsibility of the organisation (Fig. 1).

### The Training and Competencies of the Facilitator

According to the facilitators in our study, thorough training and practical experience contribute to the facilitator’s confidence and ability to create a wondering mode and positive moral inquiry in the MCD session for enhanced mutual understanding as a key endpoint of a high-quality session. Walker (1993) states that focusing on values and norms by sharing personal perspectives within the group contributes to meaningful ethical reflections as well as a collaborative approach to addressing complex moral dilemmas. When structuring the MCD session, the facilitators have some helpful suggestions. Comprehensive training, particularly in stepwise methods such as the dilemma method for MCD, plays a vital role in enabling facilitators to confidently guide dialogues within the group (Stolper et al., 2016). However, there is a delicate balance to strike between providing a structured approach that ensures focus, critical reflection, and in-depth conversation and allowing for a free, open dialogue without reducing moral deliberation to a mere checklist. Our study also emphasises the discipline of focusing on a single moral case and question during MCD sessions, which helps maintain a clear and structured approach to analysing and scrutinising the specific moral issue. This approach strikes a balance between allowing for participant interaction, which includes acknowledging emotional aspects while maintaining a task-oriented focus on the moral issue at hand, as described by Spronk et al. (2021).

Besides, facilitating clinical ethics support can be demanding (Antonsen et al., 2018). Karlsen et al. (2018) and Magelssen et al. (2016) indicate that facilitators’ characteristics wield a substantial influence on the functioning of the MCD group. The facilitator’s position requires certain leadership skills, such as active listening and tuning into the atmosphere of the group (Grönlund et al., 2019). Participants also recognise the skills of the facilitator as crucial for the quality of the group reflection session (Wocial et al., 2023). Desired competencies perceived by MCD participants are, for instance, ethical expertise, authoritarian and pedagogical leadership styles, and personal qualities such as sensitivity (Svantesson et al., 2008). Our findings show that facilitators adhere to exacting personal and professional standards, embodying qualities of attentiveness such as empathy and patience with proficient leadership and competent communication. Creating a positive atmosphere within MCD sessions emerges as one of the critical endeavours (Haan et al., 2018). On the other hand, the ability to show openness plays a pivotal role in nurturing a constructive ambience and instilling a shared sense of responsibility among participants (Weidema et al., 2015). Furthermore, in addition to shaping an inclusive environment where everyone feels safe to express their views and is treated with respect, facilitators’ experience and character are essential (Fig. 1). For facilitators in the MCD sessions, the self-awareness of capacities and knowledge of how to facilitate MCD is helpful.

### Commitment and Respect from the MCD Participants

Although the facilitators in our study did not explicitly emphasize the trait of openness within themselves, they stressed the importance of openness among all participants. The emphasis on openness encourages active engagement, fosters an environment where thoughts can be openly shared, and promotes respectful collaboration among participants. Engaging in a wondering mode during sessions becomes challenging for participants when disruptions occur during the session (Grönlund et al., 2019). It is equally challenging for the facilitator when the group consists of reluctant participants who would rather devote their time to work tasks than participate in an MCD session (Weidema et al., 2015). Unmotivated participants are perceived as disrespectful to both the facilitator and the group. Also, facilitators in our study were concerned that the participants seemed to not to fully grasp the specific characteristics and objectives of the MCD sessions. Weidema et al. (2015) described that participants felt that engaging in MCD did not concern them. In such cases, MCD facilitators invest additional time during the session to assist participants in comprehending the essence of open and reflective dialogue, along with the associated rules of conduct. For future research, it would be useful to reflect upon how MCD facilitators can better inform MCD participants of the meaning of an MCD session before the session begins.

### Responsibility of the Organisation

Nurturing the development of healthcare professionals’ moral competence requires robust organisational support (Devik et al., 2020). Our findings underscore the importance of organisational arrangements, management support, and dedicated physical spaces for MCD meetings (Fig. 1). Within an organisation, there is a need to shape a space for moral reflection (Walker, 1993), along with providing the necessary resources for training MCD facilitators (Antonsen et al., 2018; Hognestad Haaland et al., 2021). Implementing MCD in the workplace as part of a larger organisation’s integrated policy to stimulate ethics support is crucial in facilitating high-quality MCD sessions. Furthermore, MCD sessions should not be held only because the MCD facilitators enjoy MCD or have received training. Co-ownership and actively monitoring stakeholders’ experience of the usefulness of MCD is crucial for positive implementation (Weidema et al., 2016).

### Limitations

It is a methodological weakness that we mixed different data-collection sources. In hindsight, we see that a major limitation was not using telephone interviews instead of a survey. For instance, the question of difficulties would be more suitable for an interview by allowing for follow-up questions. A risk might have been that Scandinavian data would permeate the findings. Notably, while the Scandinavian data were qualitatively richer, the Dutch provided more data. Furthermore, we found as many representative quotes from Dutch as Scandinavian respondents. However, we should have complemented the study with a sample of Dutch telephone interviews. Additionally, another weakness is a lack of rigour when constructing the questionnaire which could have been addressed by piloting the study more thoroughly. Nonetheless, despite these limitations, it should be noted that the Euro-MCD evaluation instrument used in this project has been robustly validated (de Snoo-Trimp et al., 2019, 2020; Svantesson et al., 2014).

Due to the ongoing nature of the larger study, a convenient approach had to be adopted, and the study design was developed incrementally. Additionally, as this study represents the first assessment of facilitators’ perspectives from three different ethics support settings, a predefined format could not be utilised. However, these limitations will be seriously considered in future evaluation research to improve the methodological approach and address any potential biases.

## Conclusion

In conclusion, this study makes significant contributions to our understanding of how to achieve high-quality MCD. The findings highlight three essential prerequisites for fostering a morally safe space within MCD: trained virtuous facilitators, committed and respectful participants, and organisational support in terms of time and dedicated meeting locations. Building on these prerequisites, valuable insights are provided for the training of MCD facilitators, emphasising the need to enhance their moral competence, dialogical skills, and confident management abilities. Furthermore, this study emphasises the importance of leadership commitment from managers in preparing MCD participants for engaging in dialogues during moral inquiries.

The implications of this research extend to the broader field of healthcare ethics, suggesting the significance of understanding MCD dynamics to inform the selection of preferred facilitator styles and conversation methods. By incorporating these findings, healthcare organisations can take concrete steps to promote high-quality MCD practices and enhance decision-making processes. Moving forward, future research should delve deeper into the specific strategies and training interventions required to develop the competencies of MCD facilitators and foster effective participant engagement. Additionally, investigating the long-term impact of high-quality MCD on ethical decision-making and patient outcomes would provide valuable insight for healthcare professionals and organisations.
